# Enabling Nature-Based Solutions to Build Back Better—An Environmental Regulatory Impact Analysis of Green Infrastructure in Ontario, Canada

**DOI:** 10.3390/buildings12010061

**Published:** 2022-01-08

**Authors:** Vidya Anderson, William A. Gough

**Affiliations:** 1 Climate Lab, Department of Physical and Environmental Sciences, University of Toronto Scarborough, Toronto, ON M1C 1A4, Canada; 2 Department of Physical and Environmental Sciences, University of Toronto Scarborough, Toronto, ON M1C 1A4, Canada; william.gough@utoronto.ca

**Keywords:** build back better, climate change, environmental policy, green infrastructure, resilience planning, UN SDGs

## Abstract

The application of green infrastructure in the built environment delivers a nature-based solution to address the impacts of climate change. This study presents a qualitative evidence synthesis that evaluates policy instruments which enable the use and implementation of green infrastructure, using Ontario, Canada as a case study. Unpacking the elements of the policy landscape that govern green infrastructure through environmental regulatory impact analysis can inform effective implementation of this nature-based solution and support decision-making in public policy. This environmental regulatory impact analysis is based on a systematic review of existing policy instruments, contextual framing in a continuum of coercion, and identification of alignment with relevant UN SDGs. Enabling widespread usage of green infrastructure in the built environment could be a viable strategy to build back better, localize the UN SDGs, and address multiple climate change impacts.

## 1. Introduction

Capitalizing on government spending and stimulus packages to redesign communities is not a new concept, however, the current scope and scale of (re)investment is unprecedented [1,2]. The recent global novel corona virus (COVID-19) pandemic has underscored the opportunities for a green recovery. While the impacts of COVID-19 on communities around the world have been influenced by the state of healthcare infrastructure and pandemic preparedness, government response, and governance frameworks, strategic investments in nature-based solutions can support economic recovery and increase the resilience of built environments to climate change. More recently, the concept of ‘building back better’ to incorporate climate resilience and sustainability as part of community restoration and redevelopment, have become common goals [1,2,3]. This concept was recognized in the United Nations’ Sendai Framework for Disaster Risk Reduction, enacted in 2015 and adopted by member states as one of four framework priorities including disaster recovery, risk reduction, and sustainable development [4]. Such strategies and adjustments occur within the policy landscape through the use of different policy instruments that can facilitate or inhibit implementation.

Green infrastructure delivers a nature-based solution to build back better, support sustainable development, and address the impacts of climate change. Nature-based solutions have been defined by the International Union for Conservation of Nature as “actions to protect, sustainably manage, and restore natural or modified ecosystems, that address societal challenges effectively and adaptively, simultaneously providing human well-being and biodiversity benefits” [5]. Nature-based solutions provide an overarching framing of five categories of ecosystem-based approaches of which green infrastructure is one [5,6,7,8]. Green infrastructure provides various ecosystem services through interlinked networks of engineered and natural green space [8,9,10]. As a cross-sectoral approach, green infrastructure can address the impacts of climate change, with multiple environmental and health co-benefits for communities [9]. Green infrastructure can mitigate the impacts of climate change by increasing energy efficiency in the built environment, decreasing air temperature, and reducing urban heat island effect [11,12,13,14]. Studies have also shown that green infrastructure applications can mitigate atmospheric environmental pollutants and greenhouse gases [8,15,16,17,18,19,20]. Green infrastructure can also provide flood attenuation during extreme rainfall events, while improving water quality through sediment erosion control and reduced nutrient loading.

In addition to increasing health and environmental equity, green infrastructure supports implementation of the United Nations Sustainable Development Goals (UN SDGs) to build back better across communities [9]. The UN SDGs were established to eliminate poverty, safeguard the planet, and improve quality of life, globally. The 17 Goals have been adopted by every UN member state, including Canada [3]. Each UN SDG has associated targets (169) and indicators (230) with linkages and interdependencies between each of the goals [21]. Green infrastructure provides a mechanism to localize the UN SDGs in communities and build back better. Building back better to achieve the UN SDGs requires a strategic policy approach to ensure integration between the economic, environmental, and social aspects of sustainable development. While the utility of green infrastructure as a nature-based solution has been recognized by the international community and it has been acknowledged as a priority on both the Canadian federal and provincial climate change and sustainable development policy agendas, the specifics regarding implementation can be vague. The varied use of policy instruments that enable green infrastructure implementation is best understood through an analysis of regulatory impacts which are presented in this qualitative evidence synthesis.

### Literature Review

Although the benefits of green infrastructure are recognized, the pace of mainstream uptake has been slow due to upfront costs for design and construction, and conservation of natural systems [14]. Additional barriers to implementation include patchy regulatory frameworks, lack of community engagement, and the perception of green infrastructure as a principally stormwater management tool [9,10,22,23,24,25,26].

While the initial required resources for green infrastructure can exceed those for more conventional methods, stormwater and wastewater management cost reductions can be realized in avoiding grey infrastructure construction, reducing sewage treatment costs, and avoided flood losses support the initial investment [14]. In addition, green infrastructure provides ecosystem services necessary for human health and well-being including clean drinking water, breathable air, food, climate change mitigation, and natural resources for a range of human activities. Globally, ecosystem services are valued at approximately USD 125T which annually support farming, fishing, forestry, and tourism industries with over a billion people employed [27]. Global loss of ecosystem services due to land use change is valued at USD 4–20T annually with ecosystem services contributing more than twice as much to human well-being as global gross domestic product (GDP) [27] and more than 50 percent of global GDP is dependent on nature and its ecosystem services [28].

Investment in green infrastructure can also result in direct and indirect health co-benefits within the built environment. One concerning example is the increasing occurrence of algae blooms on lakes and the attendant risk of exposure to cyanobacteria resulting from human induced eutrophication of water bodies. Green infrastructure such as tree-based intercropping systems can ameliorate water quality in lakes and rivers, by reducing reliance on pesticides and fertilizers commonly used in conventional agricultural systems [29,30]. Green infrastructure applications have improved respiratory health conditions resulting from air pollutants and extreme heat [8,10,14,31,32,33,34,35,36,37,38,39]. For example, green-roofing and green wall technologies can reduce air pollutant concentrations and provide urban cooling [8,10,14,39,40,41]. Patients recovering from surgery have benefited from exposure to vegetation and forestry [42]. The urban forests in the United States have impacted air pollution and this has been valued at USD 4B with the abatement of 711,000 metric tonnes of pollutants annually [31,43] while trees in 86 Canadian cities removed 16,500 tonnes of air pollution with resulting human health benefits valued at approximately CAD 227M [38].

Human panel studies have demonstrated that exposure to vegetation and forestry can produce positive health benefits [44,45,46]. Residential green infrastructure has been linked to reduced mortality from cardiovascular, respiratory, and other causes in various cohort studies [47,48,49,50]. Green infrastructure reduces the risk of spreading of infectious disease by providing suitable habitats for vector and zoonotic reservoir populations [43]. While landscape fragmentation can amplify disease spread in human and animal populations, green infrastructure can behave as a barrier [43,51,52,53,54]. Such benefits are not insignificant, when building back better within the post-pandemic landscape.

**Figure 1:**
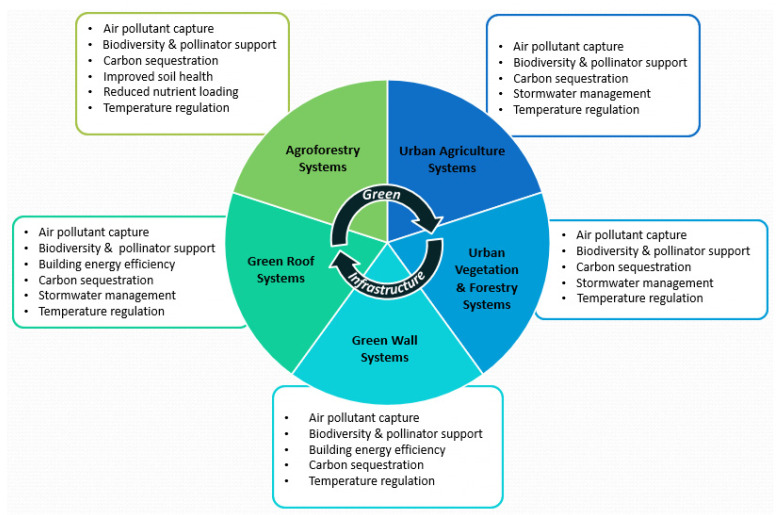
Green infrastructure form and function [8,9,10,14,39,60].

Socioeconomic systems are unpinned by natural systems. Green infrastructure can increase real estate valuation, while reducing air pollution, electricity, and stormwater management costs [11,12,13,31,38,43,55,56,57,58,59]. Using green infrastructure as a nature-based solution to promote healthy built environments and maintain healthy ecosystems, can support resilience to the socioeconomic impacts of multiple externalities. Green infrastructure has demonstrated economic and health benefits [8,10,14,39,60]. These benefits need consideration within the broader context of climate change. The application of green infrastructure does provide a pathway for addressing climate change; however, each application is a complex intervention with specific characteristics that can be utilized effectively if strategically applied. These are illustrated in Figure 1.

As previously noted, the benefits of green infrastructure are well-established, however, mainstream implementation has been slow due to upfront design-build and land conservation costs [14]. Other implementation barriers include patchy regulatory frameworks, lack of community engagement, and a focus on green infrastructure for stormwater management [9,10,22,23,24,25,26]. Previous green infrastructure policy research has focused primarily on policy integration, stakeholder engagement, financing models; or project implementation [22,23,61,62,63,64,65]. While this work holds significant value, to achieve greater uptake of green infrastructure, it is essential to understand the individual policy instruments that enable its implementation, how they function, and the environmental outcomes they are designed to achieve. The varied use of policy instruments that enable green infrastructure implementation is best understood through an analysis of regulatory impacts which are presented in this qualitative evidence synthesis. Environmental regulatory impact analysis is a method for supporting decision and policy making by enabling implementation of nature-based solutions.

This study has sought to answer the research question of how the varied use of policy instruments that enable green infrastructure implementation can be understood through an analysis of regulatory impacts. This paper presents a regulatory impact analysis (RIA) framework, developed specifically for the examination of environmental issues (E-RIA). The authors use as a case study, legislation and resultant regulation in Ontario, Canada, to illustrate this environmental RIA framework. The authors present a model for E-RIA and apply it to the case study of Ontario, Canada. Deconstructing the elements of the policy landscape which govern green infrastructure can inform effective implementation of this nature-based solution within the built environment. E-RIA can identify areas of overlap and opportunity for greater environmental policy integration. This environmental regulatory impact analysis (E-RIA) presents a pathway for others to follow when evaluating which types of policy instruments to use in the strategic implementation of green infrastructure. The novelty of this work is in the creation of an environmental RIA framework that can be applied across other jurisdictions. As part of this work, we introduce for the first time in the peer reviewed literature, the policy continuum of coercion, using elements in common practice in public policy. The UN SDGs are also specifically included as an integral evaluative lens for undertaking an E-RIA.

## 2. Methods

The authors set out to navigate the public policy landscape in Ontario, Canada and the unique set of instruments that govern it. The research question that this study has sought to answer is how the varied use of policy instruments enabling green infrastructure implementation can be understood through an analysis of regulatory impacts. In order to navigate this landscape, it was necessary to deconstruct Ontario’s governance framework through environmental regulatory impact analysis undertaken as part of a qualitative evidence synthesis. Qualitative evidence synthesis helps to explore barriers and enablers to the delivery and uptake of services to inform their prioritization [66]. This type of review provides a thematic analysis that may include conceptual models as demonstrated in this study.

### 2.1. Theoretical Framework for Environmental Regulatory Impact Analysis

This qualitative evidence synthesis presents an environmental regulatory impact analysis (E-RIA) as a systematic framework to critically assess the positive and negative effects of proposed and existing regulations and non-regulatory alternatives. The practice of regulatory impact analysis (RIA) is used across member countries of the Organization for Economic Co-operation and Development (OECD) and encompasses a range of methods. The OECD was established in 1960 when 18 European countries, in addition to Canada and the United States, came together to promote economic development through good governance. RIA is an important element of an evidence-based approach to policy making and program implementation. OECD analysis indicates that performing RIA can strengthen the capacity of governments to ensure that regulations are effective, efficient, and responsive to the complexity of a changing world [67]. RIA is a method designed to support policy coherence by improving the use of evidence in policy making.

**Figure 2:**
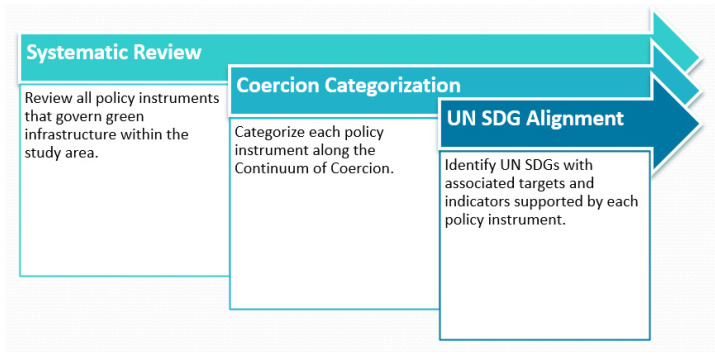
E-RIA methodological pathway.

The practice of RIA is widely used across all OECD jurisdictions, but application varies widely between nations and levels of government. Examples of completed RIAs are rarely if ever published by governments due to the confidential nature of the circumstances in which RIAs tend to be undertaken. One rare, published example of an RIA was undertaken by the Irish Department of Health for the Public Health (Alcohol) Bill [68]. Interestingly, while publication of completed RIAs is rare, there are many examples of RIA guidance and best practices published by various governments including Canada, Australia, Scotland, Ireland, the United Kingdom, and Sweden, among others [69]. The regulatory development process undertaken by governments writ large is semi-transparent at best although the practice of RIA is an important step toward building greater transparency and accountability. The most common form of RIA is used as part of the development process for new government policies and regulations and is considered to be ex-ante analysis [70]. Ex-ante RIAs are undertaken in advance of drafting new legislation to troubleshoot regulatory outcomes. The ex-post form of RIA occurs after legislation has been in enacted to gauge its efficacy. This form of RIA is rarely undertaken but holds significant value as it enables an evaluation of the real-time impacts of policy and regulation after they are in force [71]. This review paper presents an ex-post form of environmental regulatory impact analysis conducted outside of government. To support this environmental regulatory impact analysis, the following methodological pathway was undertaken as shown in Figure 2.

The first step in the ex-post E-RIA pathway was to conduct a systematic review of all existing policy instruments within the study area or jurisdiction. The second step was to categorize each policy instrument along a continuum of coercion that illustrates different levels of government control over specific activities. The third step in the methodological pathway was to identify the individual UN SDGs supported by each policy instrument and the associated targets and indicators.

### 2.2. Systematic Review

**Figure 3:**
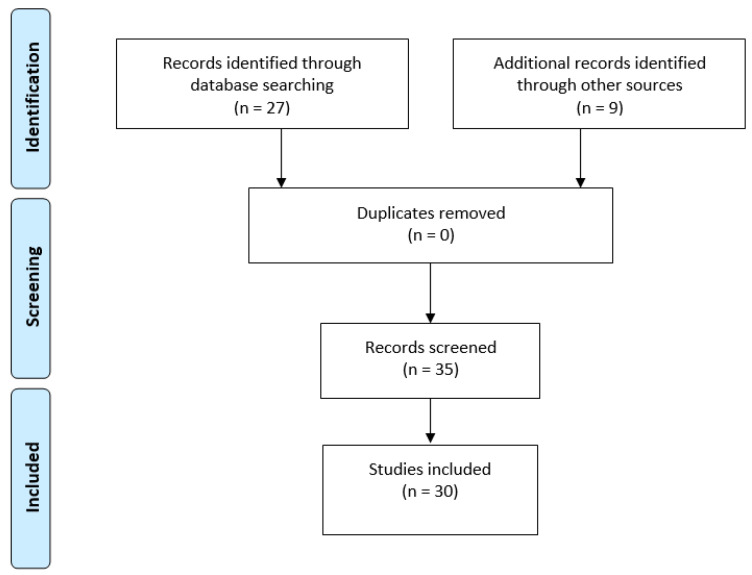
Overview of studies identified in the steps of the systematic review process derived from the PRISMA flow diagram [72].

A systematic review of individual policy instruments was undertaken to identify relevant regulations, standards, guidelines, and programs specific to the jurisdictional case study of Ontario, Canada for the application of green infrastructure. Regulations, standards, guidelines, grant funding opportunities, and program materials such as strategies or fact sheets were screened and based on relevance full documents were retrieved to determine inclusion within the review using variations on the search term “green infrastructure” and “nature-based solutions”. As illustrated in Figure 3, a total of 30 relevant policy instruments were identified for inclusion in the regulatory impact analysis.

### 2.3. The Policy Continuum of Coercion

The application of green infrastructure in Ontario, Canada is directed by a unique set of instruments within the public policy landscape. To better navigate this landscape, it is necessary to unpack its governance framework. Public policy is set by those who have the legal authority to establish and enforce standards of normative behaviour. Policy is influenced by various actors, issues, interests, and circumstances. Put simply, policy is a decision and subsequent suite of actions, developed and implemented to address a need, problem, or issue.

**Figure 4:**
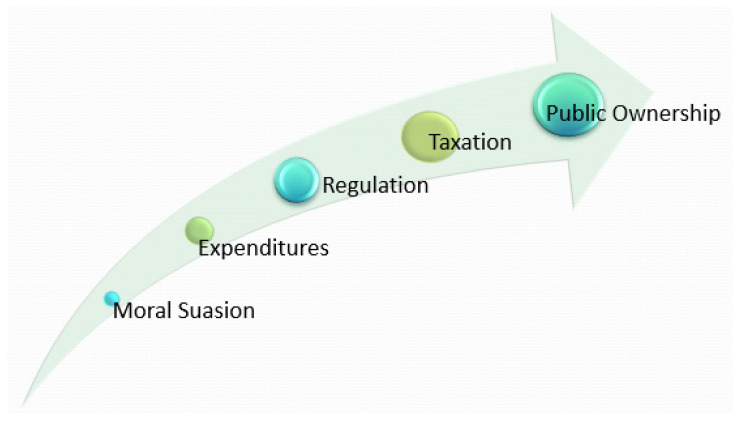
The Policy Continuum of Coercion [39].

The concept of public policy is usually linked with the rule of law and the passage of legislation, but it is not limited to these vehicles. Policy objectives are achieved using different policy instruments. The choice of instrument is determined by the desired level of coercion or control in achieving an objective. Figure 4 illustrates the continuum of coercion across the various instruments available in the policy making process. Policy instruments can be used to instill behaviour change, influence socioeconomic conditions, and provide public services. Factors in instrument choice include the political climate, in addition to fiscal and social constraints.

The policy continuum of coercion illustrates different levels of government control over specific activities. Moral suasion instruments are the least coercive, exhorting or admonishing the target group to pursue or cease a particular action. For example, many jurisdictions will ask residents to limit all non-essential water use such as car-washing during heatwaves, in order to conserve and maintain adequate water levels for emergencies such as firefighting. Other moral suasion examples include alcohol and tobacco cessation advertising campaigns illustrating the deleterious health effects of consumption. Expenditures are low-level instruments of coercion that seek to incentivize behaviour change through financial means. For example, many jurisdictions will offer a rebate for energy efficiency retrofits undertaken on a residence to reduce greenhouse gas emissions [73]. Eligible retrofits may include installation of new windows, energy efficient appliances, solar panels, or high efficiency furnaces. Other examples include grant programs in flood prone areas to reduce repetitive flood risk to building infrastructure [74]. Regulation is a more coercive instrument. For example, many jurisdictions prohibit dumping of chemicals, toxic waste, or garbage into rivers, lakes, or other marine environments, and will impose heavy fines and penalties for violations. Other examples include imposing vehicular speed limits and requiring the use of seatbelts when driving. Taxation is a very coercive instrument that seeks to discourage certain behaviours by imposing a financial burden on the targeted activity. For example, some jurisdictions have implemented a congestion charge to reduce vehicular traffic and air pollution in urban centres [75,76]. Other examples of heavily taxed commodities include alcohol and cigarettes. Public ownership is the most coercive of all policy instruments wherein the state will take on the ownership and administration of a particular activity in order to maintain complete control over it. Examples include the production and distribution of crude oil, and the generation, distribution and sale of electricity.

There are fiscal and political implications for each type of instrument along the policy continuum of coercion. Moral suasion and expenditure instruments require the government to have the fiscal resources to dedicate to their development. These types of instruments may be more desirable for decision-makers if public appetite for state intervention in the form of regulation or taxation is low. Public ownership requires both fiscal capacity and significant state intervention to undertake the governance, oversight, and administration of an activity. Use of the different types of instruments along the continuum will vary with the political appetite and political climate within each state or jurisdiction.

### 2.4. The UN SDG Evaluative Lens

The UN SDGs were established to eliminate poverty, safeguard the planet, and improve quality of life, globally as shown in Table 1. Each UN SDG has associated targets (169) and indicators (230) with linkages and interdependencies between each of the goals. As part of the E-RIA model, we use the UN SDGs as an evaluative lens to determine the environmental outcomes each policy instrument is designed to achieve.

## 3. Results

Green infrastructure in Ontario, Canada is governed by 30 policy instruments along the continuum of coercion. Across the continuum, the policy instruments which enable the implementation of green infrastructure in Ontario, Canada include moral suasion, expenditures, regulation, taxation, and public ownership as illustrated in Figure 4. There is no integrated public policy on green infrastructure in Ontario, Canada per se, rather there is a patchwork of instruments that address green infrastructure within varying contexts. Although not integrated through a central policy or strategy, this range of policy instruments supports the localization of specific UN SDGs as shown in Table 1.

### 3.1. Moral Suasion

Moral suasion is the least coercive instrument used as part of green infrastructure public policy with the greatest variation and interpretation. In Ontario, there are thirteen instruments of moral suasion which include the Ontario Climate Change Action Plan, Ontario’s Climate Change Strategy, A Made-in-Ontario Environment Plan, Great Lakes Strategy, the Green Belt Plan, the Growth Plan for the Greater Golden Horseshoe, the Low Impact Development (LID) Stormwater Management Guidance Manual, the Niagara Escarpment Plan, the Oak Ridges Moraine Conservation Plan, the Provincial Policy Statement, municipal official plans, the Stormwater Management Planning and Design Manual, and the Wetland Conservation Strategy.

The Ontario Climate Change Action Plan, Ontario’s Climate Change Strategy, and A Made-in-Ontario Environment Plan are low level instruments of coercion. Each document is descriptive and aspirational and loosely sets out different iterations of the provincial government’s climate change policy. Green infrastructure is referenced as a solution to restore ecosystems, reduce atmospheric carbon, and protect and expand carbon sinks [77]. The importance of green infrastructure is also highlighted in lowering greenhouse gas emissions, reducing pollution, and helping to make community infrastructure more resilient [78]. Each of these instruments specifically supports UN SDG 13—Climate Action to take urgent action to combat climate change and its impacts; UN SDG 11—Sustainable Cities and Communities to make cities and human settlements inclusive, safe, resilient, and sustainable; and UN SDG 15—Life on Land to protect, restore, and promote sustainable use of terrestrial ecosystems, sustainably manage forests, combat desertification, and halt and reverse land degradation and halt biodiversity loss.

The Great Lakes Strategy and the Wetland Conservation Strategy are the least coercive of the thirteen moral suasion instruments. These two strategies provide a vision, goals, and priorities to help restore, protect, and conserve the Great Lakes and Ontario’s wetlands [79,80]. These documents are descriptive and aspirational. Green infrastructure is mentioned 15 times in the Great Lakes Strategy as a source control measure to reduce stormwater volumes, mitigate nutrients, create habitat, and enhance biodiversity [78]. Within the Wetland Conservation Strategy, green infrastructure is mentioned seven times as a means to improve air and water quality, manage stormwater, reduce flood impacts, decrease energy use, and increase carbon storage in vegetation. These instruments specifically support UN SDG 15—Life on Land to protect, restore, and promote sustainable use of terrestrial ecosystems, sustainably manage forests, combat desertification, and halt and reverse land degradation and halt biodiversity loss.

Moving through the suite of thirteen moral suasion instruments, the Low Impact Development (LID) Stormwater Management Guidance Manual and the Stormwater Management Planning and Design Manual, provide best management practices for managing stormwater in Ontario. Used in conjunction, these manuals enable and promote design alternatives using green infrastructure applications instead of or in addition to conventional grey infrastructure to manage stormwater at the source, in addition to reducing runoff and nutrient loading. This instrument specifically supports UN SDG 11—Sustainable Cities and Communities to make cities and human settlements inclusive, safe, resilient, and sustainable.

The remaining instruments of moral suasion work together to provide policy direction on all land use planning and development matters in Ontario, Canada. These instruments support the application of green infrastructure as a sustainable development tool. The Provincial Policy Statement sets out Ontario’s priorities within the land use planning system and works in conjunction with the Greenbelt Plan, the Niagara Escarpment Plan, the Oak Ridges Moraine Conservation Plan, the Growth Plan for the Greater Golden Horseshoe, and all municipal official plans [81]. Green infrastructure is mentioned five times in the Provincial Policy Statement as a form of infrastructure which complements conventional grey infrastructure [81]. The Provincial Policy Statement supports UN SDG 11—Sustainable Cities and Communities to make cities and human settlements inclusive, safe, resilient, and sustainable. It also supports UN SDG 13—Climate Action to take urgent action to combat climate change and its impacts.

The Greenbelt Plan works together with the Oak Ridges Moraine Conservation Plan and the Niagara Escarpment Plan to identify areas within the agricultural land base, in addition to ecological and hydrological features, areas and functions occurring in the landscape to be permanently protected from urbanization and development [82]. The Growth Plan for the Greater Golden Horseshoe is a long-term plan that works in conjunction with the Greenbelt Plan, the Oak Ridges Moraine Conservation Plan, and the Niagara Escarpment Plan to provide a framework for urbanization and growth management across the region [83]. The Oak Ridges Moraine Conservation Plan, the Niagara Escarpment Plan, and the Growth Plan for the Greater Golden Horseshoe collectively mention green infrastructure 24 times as a means of increasing climate resilience, reducing risk to life and property, decreasing the incidence of repair or replacement resulting from extreme weather events, and capturing and treating runoff from impervious surfaces. These instruments support UN SDG 11—Sustainable Cities and Communities to make cities and human settlements inclusive, safe, resilient, and sustainable. They also support UN SDG 13—Climate Action to take urgent action to combat climate change and its impacts and UN SDG 2—Zero Hunger to end hunger, achieve food security and improved nutrition and promote sustainable agriculture.

In Ontario, Canada, a municipal official plan sets out a municipality’s general land use planning policy. It provides a vision, goals, priorities, and guidance for how land in a community should be used. Municipalities can choose to prioritize the use and application of green infrastructure within a community through their official plans. A scan of 93 municipal official plans across Ontario shows that approximately 23 official plans specifically mention green infrastructure in the context of climate resilience and stormwater management. These instruments support UN SDG 11—Sustainable Cities and Communities to make cities and human settlements inclusive, safe, resilient, and sustainable, in addition to supporting UN SDG 13—Climate Action to take urgent action to combat climate change and its impacts.

### 3.2. Expenditures

Across the continuum of coercion in the province of Ontario, expenditures are used to enable the application of green infrastructure at the local level as evidenced by the Eco-Roof Incentive program administered by the City of Toronto and a range of grants administered by the province of Ontario and the Federal Government of Canada. This includes the Clean Water and Wastewater Fund which is co-funded by the federal and provincial government and administered by the province; and the federal Green Infrastructure Phase II suite of programs [84]. Nomenclature used within these programs varies from specific green infrastructure applications such as green and white roofs in the Eco-Roof Incentive program, to less obvious terms such as stormwater management in the Clean Water and Wastewater program, and technologies to reduce water use and impacts on aquatic ecosystems in the Clean Growth program (part of the federal Green Infrastructure Phase II suite of programs). Federally, the Canadian Government has supports green infrastructure using a wide range of terms (e.g., natural infrastructure, climate resilient infrastructure, and stormwater management technology) as a priority under its CAD 180B infrastructure plan—Investing in Canada [84]. Green infrastructure has been highlighted as a means to preserve the health of the environment and to promote sustainable and healthy community development [84]. Additionally, the Investing in Canada Infrastructure Program has allocated CAD 27B in federal infrastructure funding to green infrastructure for provinces and territories to facilitate the reduction of GHG emissions; enable greater resilience and adaptation to climate change impacts and climate-induced disaster mitigation; and to ensure the provision of clean air and safe drinking water [84]. The federal government has also established a CAD 4B Natural Climate Solutions Fund that supports planting two billion trees; restoring and enhancing wetlands, peatlands, and grasslands to store and capture carbon; and implementing farming practices to tackle climate change [85]. The federal government has invested a further CAD 225M in a National Disaster Mitigation Program for provinces and territories to build safer and more resilient communities through mitigation investments including green infrastructure that could reduce, or even negate, the effects of flood events [86]. These expenditure instruments support UN SDG 11—Sustainable Cities and Communities to make cities and human settlements inclusive, safe, resilient, and sustainable. They also support UN SDG 13—Climate Action to take urgent action to combat climate change and its impacts.

### 3.3. Regulation

Moving along the continuum of coercion, regulation has been used to enable the application of green infrastructure in Ontario, Canada. The interpretation of green infrastructure is variable across the eight regulatory instruments and the context for its application varies. The Great Lakes Protection Act and the Nutrient Management Act enable the application of green infrastructure to control point source pollution and reduce runoff and nutrient loading. The Great Lakes Protection Act works in conjunction with the Great Lakes Strategy and the prime function in the application of green infrastructure is to protect and restore the ecological health of the Great Lakes and increase resilience to growth and development pressures [87]. These instruments work together to support UN SDG 15—Life on Land to protect, restore, and promote sustainable use of terrestrial ecosystems, sustainably manage forests, combat desertification, and halt and reverse land degradation and halt biodiversity loss.

Regulation 267/03 under the Nutrient Management Act requires the application of green infrastructure specifically to manage runoff from agricultural fields to water bodies through the application of riparian buffers and buffer strips comprised of trees, shrubs, or vegetation [88]. The Planning Act enables the application of green infrastructure in broad terms to protect the agricultural land base, natural resources, and the environment writ large by promoting sustainable growth and urbanization [89]. These instruments support UN SDG 11—Sustainable Cities and Communities to make cities and human settlements inclusive, safe, resilient, and sustainable. They also support UN SDG 15—Life on Land to protect, restore, and promote sustainable use of terrestrial ecosystems, sustainably manage forests, combat desertification, and halt and reverse land degradation and halt biodiversity loss.

The Ontario Building Code governs the construction of new buildings and the renovation of existing buildings. The Code sets minimum standards of construction for built structures and establishes a uniform set of building standards for the province. The Code enables application of green infrastructure by allowing for the installation of vegetative roof surfaces [90]. The Municipal Act and the City of Toronto Act set out the administrative, financial, and legislative powers of municipalities in Ontario, Canada. Both the Municipal Act and the City of Toronto Act enable the application of green infrastructure across municipalities by, (a) authorizing municipalities to adopt and maintain plans requiring the protection and enhancement of tree canopy and natural vegetation; and (b) allowing municipalities to pass green roof by-laws [91]. These instruments work together to support UN SDG 11—Sustainable Cities and Communities to make cities and human settlements inclusive, safe, resilient, and sustainable.

The Toronto Green Standard sets out sustainable design requirements for new private and public developments specifically in the City of Toronto, which is the largest municipality and the capital city of the province of Ontario [92]. The standard is comprised of tiered levels of performance measures and guidelines to promote sustainable design for sites and buildings. The standard enables the application of green infrastructure to address the City of Toronto’s environmental pressures which include improving air quality; reducing urban heat island effect; reducing storm water runoff and improving the quality of storm water draining into Lake Ontario, protection and enhancement ecological functions, and integration of landscapes and habitats on site [91]. This instrument supports UN SDG 11—Sustainable Cities and Communities to make cities and human settlements inclusive, safe, resilient, and sustainable. The standard also supports UN SDG 15—Life on Land to protect, restore, and promote sustainable use of terrestrial ecosystems, sustainably manage forests, combat desertification, and halt and reverse land degradation and halt biodiversity loss.

### 3.4. Taxation

The three taxation instruments used to enable the application of green infrastructure include development charges, municipal property taxes, and municipal stormwater fees. The context for the application of green infrastructure for these taxation instruments is stormwater management. Development charges are used by municipalities under the authority provided by the Development Charges Act, to finance the cost of installing infrastructure to support new development. Development charges are often used to finance conventional stormwater infrastructure but can include green infrastructure. Municipal property taxes are used to finance municipal stormwater management among other municipal services such as firefighting, policing, transit, and roads. Stormwater can be managed using both conventional grey infrastructure and green infrastructure depending on a municipality’s sustainability objectives. The last taxation instrument is municipal stormwater fees which are imposed by a municipality on property owners under the authority of the Municipal Act in order to finance the cost of managing stormwater. Stormwater fees can be used to incentivize the application of green infrastructure to manage stormwater onsite and reduce runoff. These taxation instruments support UN SDG 11—Sustainable Cities and Communities to make cities and human settlements inclusive, safe, resilient, and sustainable.

### 3.5. Public Ownership

The final policy instrument from the continuum of coercion used to enable the application of green infrastructure is public ownership. The Infrastructure for Jobs and Prosperity Act. Regulation 588/17 under the Infrastructure for Jobs and Prosperity Act requires each municipality in Ontario, Canada to adopt an asset management plan to ensure the sustainability of municipal infrastructure assets, including green infrastructure, over the long-term. Green infrastructure assets within the regulation include various applications such as natural features and systems, parklands, street trees, urban forests, natural channels, and green roofs [93]. This instrument supports UN SDG 11—Sustainable Cities and Communities to make cities and human settlements inclusive, safe, resilient, and sustainable. It also supports UN SDG 15—Life on Land to protect, restore, and promote sustainable use of terrestrial ecosystems, sustainably manage forests, combat desertification, and halt and reverse land degradation and halt biodiversity loss.

## 4. Discussion

Environmental policy in Ontario, Canada utilizes all the policy instruments on the continuum of coercion, however, the policy landscape that governs the adoption and implementation of green infrastructure within the built environment is not integrated. Although not explicitly revealed by the coercion and UN SDG analyses, this review of the policy instruments shows there is no central strategy or document that coordinates or integrates green infrastructure policy instruments all together within this jurisdiction. This is not to say the existing instruments are ineffectual for the purpose for which they were designed, however, having a central strategy or even a stated position within the larger public policy agenda on the use of green infrastructure would provide greater coherence in its use and enable wider implementation. Each of the different policy instruments addresses the use of green infrastructure within different contexts but the instruments are not designed to complement each other, only to deal with a specific set of circumstances. As shown in Table 2, the different policy instruments that govern the implementation of green infrastructure, range across the continuum of coercion and support specific UN SDGs.

Although there is no integrated public policy for green infrastructure implementation in Ontario, Canada, there are explicit linkages between Ontario’s climate change policy and green infrastructure writ large. Ontario’s Climate Change Strategy has explicitly indicated that green infrastructure is a solution to aid in the restoration of ecosystems, the reduction atmospheric carbon, and the protection and expansion of carbon sinks [77]. The strategy also indicated the need to develop a multi-faceted approach to reduce emissions from urban infrastructure, existing and new, and to integrate climate change adaptation priorities into decision-making [77]. In addition, the Ontario Climate Change Action Plan points to the carbon sequestration in agricultural and natural systems and the incentivization of low-carbon communities as key priorities [94]. Following a change in the provincial government in Ontario, Canada in 2018, the Government issued A Made-in-Ontario Environment Plan designed to address environmental challenges, including climate change, and highlights the importance of green infrastructure in lowering greenhouse gas emissions, reducing other pollutants, and enhancing community resiliency [78].

At the national level, the Healthy Environment and a Healthy Economy is Canada’s Climate Plan that identifies green infrastructure as a core component under its ‘natural climate solutions’ pillar [95]. The plan focuses on restoring and conserving nature to strengthen climate benefits by planting trees, conserving and restoring ecosystems, and improving management of lands and waters. The plan makes dedicated financial investments in green infrastructure to protect nature and accelerate the sequestration potential of the natural environment across Canada’s provinces and territories [95].

The Intergovernmental Panel on Climate Change (IPCC) has highlighted green infrastructure as a means for enhancing urban carbon sinks and undertaking ecosystem-based adaptation that will transform the built environment through phytoremediation [96]. In addition, the IPCC further indicates that green infrastructure, including both green roofs and tree-based intercropping systems, have the potential to synergize mitigation and adaptation [97]. The International Union for Conservation of Nature promotes green infrastructure as a means to protect biodiversity and to enhance nature’s ecosystem services [98,99]. In America, the US Environmental Protection Agency narrowly defines green infrastructure in the Clean Water Act as a mechanism to address stormwater issues [100].

Undertaking an environmental regulatory impact analysis (E-RIA) to critically assess the positive and negative effects of regulations and non-regulatory alternatives is an important element of strategic public policy making. Using the E-RIA model to evaluate policy instruments can strengthen the capacity of governments to ensure that environmental regulations are effective, efficient, and responsive to the complexities of a changing world. As shown in Figure 2, there is a robust suite of policy instruments that govern the implementation of green infrastructure in Ontario, Canada. Green infrastructure in Ontario, Canada is governed by a unique set of instruments that span the policy continuum of coercion. Although the governance framework is not integrated by a central strategy, there are a range of policy instruments available to support implementation of green infrastructure as a nature-based tool for building back better in support of the UN SDGs.

The E-RIA model presents an opportunity to create greater policy coherence and integration; build awareness of policy gaps and opportunities; and increase fluency with the suite of policy instruments which enable green infrastructure implementation. In public policy making, tensions arise between the availability of fiscal resources, political will, and intentions to protect the environment. When implementing environmental policy, decision-makers face challenges in implementing environmental policy to achieve real outcomes and improvements without heavy state intervention. When building back better, jurisdictions will be faced with difficult choices including funding capacity, public appetite for regulation of activities, and political will to enact change. This tool enables evaluation of available policy instruments to determine if resources are required, the coercive nature of a policy instrument, and whether the instrument supports localization of the UN SDGs. Future work of interest includes applying the E-RIA model across different jurisdictions and multiple regulatory frameworks. It is of interest to explore the coercive nature of policy instruments across jurisdictions and the level of localization of UN SDGs across communities.

## 5. Conclusions

Green infrastructure has permeated public policy from the grassroots level in Ontario, Canada and the result is a varied suite of policy instruments that enable its application in the built environment. This review paper provides a map for jurisdictions to navigate the policy landscape governing the adoption and implementation of green infrastructure, using Ontario, Canada as a case study. This qualitative evidence synthesis also demonstrates a model for undertaking environmental regulatory impact assessments which is valuable given the paucity of such approaches in the peer reviewed literature. The E-RIA model can help to better understand how the UN SDGs have been localized within regulatory frameworks and the limitations of their implementation across different jurisdictions. It can also help to evaluate how policy instruments at different levels of government function and how they can be improved.

Prioritizing green infrastructure as a nature-based tool to build back better and increase climate resilience provides an opportunity to develop a centralized strategy that integrates different green infrastructure policy instruments. Undertaking an environmental regulatory impact assessment provides the ability to identify gaps, eliminate inconsistencies, and develop strategic, integrated, and coherent environmental policy for nature-based solutions.

Governments have committed funding in the order of billions to respond to the effects of the coronavirus and facilitate a global economic recovery. If rebuilding occurs in the conventional fashion with investments in fossil fuel-based growth, inequities and disparities in health, wealth, and well-being will continue to grow. Understanding the elements of the policy landscape that govern green infrastructure is essential for its effective implementation as part of economic recovery packages. This presents an unprecedented opportunity for jurisdictions around the world to build back better, increase climate resilience, and ensure a sustainable future using nature-based solutions such as green infrastructure.

## Figures and Tables

**Table 1:** The 17 UN Sustainable Development Goals.

UN Sustainable Development Goals	
**Goal 1.** No Poverty	End poverty in all its forms everywhere
**Goal 2.** Zero Hunger	End hunger, achieve food security and improved nutrition and promote sustainable agriculture
**Goal 3.** Health and Well-being	Ensure healthy lives and promote well-being for all at all ages
**Goal 4.** Quality Education	Ensure inclusive and equitable quality education and promote lifelong learning opportunities for all
**Goal 5.** Gender Equality	Achieve gender equality and empower all women and girls
**Goal 6.** Clean Water and Sanitation	Ensure availability and sustainable management of water and sanitation for all
**Goal 7.** Affordable and Clean Energy	Ensure access to affordable, reliable, sustainable, and modern energy for all
**Goal 8.** Decent Work and Economic Growth	Promote sustained, inclusive, and sustainable economic growth, full and productive employment, and decent work for all
**Goal 9.** Industry, Innovation, and Infrastructure	Build resilient infrastructure, promote inclusive and sustainable industrialization and foster innovation
**Goal 10.** Reduced Inequalities	Reduce inequality within and among countries
**Goal 11.** Sustainable Cities and Communities	Make cities and human settlements inclusive, safe, resilient, and sustainable
**Goal 12.** Responsible Production and Consumption	Ensure sustainable consumption and production patterns
**Goal 13.** Climate Action	Take urgent action to combat climate change and its impacts
**Goal 14.** Life Below Water	Conserve and sustainably use the oceans, seas, and marine resources for sustainable development
**Goal 15.** Life on Land	Protect, restore, and promote sustainable use of terrestrial ecosystems, sustainably manage forests, combat desertification, and halt and reverse land degradation and halt biodiversity loss
**Goal 16.** Peace, Justice, and Strong Institutions	Promote peaceful and inclusive societies for sustainable development, provide access to justice for all and build effective, accountable, and inclusive institutions at all levels
**Goal 17.** Partnerships for the Goals	Strengthen the means of implementation and revitalize the global partnership for sustainable development

**Table 2:** The green infrastructure policy landscape in Ontario, Canada.

Coercion Category	Policy Instrument	Corresponding UN SDG
**Moral Suasion**	Ontario Climate Change Action Plan Ontario’s Climate Change Strategy A Made-in-Ontario Environment Plan	**Goal 11—Sustainable Cities and Communities**: Make cities and human settlements inclusive, safe, resilient, and sustainable **Targets**: **11.a** Support positive economic, social and environmental links between urban, peri-urban and rural areas by strengthening national and regional development planning. **11.b** By 2020, substantially increase the number of cities and human settlements adopting and implementing integrated policies and plans towards inclusion, resource efficiency, mitigation and adaptation to climate change, resilience to disasters, and develop and implement, in line with the Sendai Framework for Disaster Risk Reduction 2015–2030, holistic disaster risk management at all levels. **11.5** By 2030, significantly reduce the number of deaths and the number of people affected and substantially decrease the direct economic losses relative to global gross domestic product caused by disasters, including water-related disasters, with a focus on protecting the poor and people in vulnerable situations. **11.6** By 2030, reduce the adverse per capita environmental impact of cities, including by paying special attention to air quality and municipal and other waste management. **11.7** By 2030, provide universal access to safe, inclusive and accessible, green and public spaces, in particular for women and children, older persons and persons with disabilities.
		**Goal 13—Climate Action**: Take urgent action to combat climate change and its impacts. **Target: 13.1** Strengthen resilience and adaptive capacity to climate-related hazards and natural disasters in all countries.
		**Goal 15—Life on Land**: Protect, restore, and promote sustainable use of terrestrial ecosystems, sustainably manage forests, combat desertification, and halt and reverse land degradation and halt biodiversity loss. **Targets**: **15.1** By 2020, ensure the conservation, restoration and sustainable use of terrestrial and inland freshwater ecosystems and their services, in particular forests, wetlands, mountains and drylands, in line with obligations under international agreements. **15.2** By 2020, promote the implementation of sustainable management of all types of forests, halt deforestation, restore degraded forests and substantially increase afforestation and reforestation globally. **15.3** By 2030, combat desertification, restore degraded land and soil, including land affected by desertification, drought and floods, and strive to achieve a land degradation-neutral world. **15.4** By 2030, ensure the conservation of mountain ecosystems, including their biodiversity, in order to enhance their capacity to provide benefits that are essential for sustainable development. **15.5** Take urgent and significant action to reduce the degradation of natural habitats, halt the loss of biodiversity and, by 2020, protect and prevent the extinction of threatened species. **15.9** By 2020, integrate ecosystem and biodiversity values into national and local planning, development processes, poverty reduction strategies and accounts.
	4. Great Lakes Strategy 5. Wetland Conservation Strategy	**Goal 15—Life on Land** **Targets**: 15.1, 15.2, 15.3, 15.4, 15.5, 15.9
	6. Low Impact Development (LID) Stormwater Management Guidance Manual 7. Stormwater Management Planning and Design Manual	**Goal 11—Sustainable Cities and Communities** **Targets**: 11.a, 11.b, 11.5, 11.6, 11.7
	8. Provincial Policy Statement	**Goal 11—Sustainable Cities and Communities** **Targets**: 11.a, 11.b, 11.5, 11.6, 11.7 **Goal 13—Climate Action** **Target**: 13.1
	9. Greenbelt Plan 10. Growth Plan for the Greater Golden Horseshoe 11. Niagara Escarpment Plan 12. Oak Ridges Moraine Conservation Plan	**Goal 2—Zero Hunger**: End hunger, achieve food security and improved nutrition and promote sustainable agriculture. **Target**: 2.4 By 2030, ensure sustainable food production systems and implement resilient agricultural practices that increase productivity and production, that help maintain ecosystems, that strengthen capacity for adaptation to climate change, extreme weather, drought, flooding and other disasters and that progressively improve land and soil quality. **Goal 11—Sustainable Cities and Communities** **Targets**: 11.a, 11.b, 11.5, 11.6, 11.7 **Goal 13—Climate Action** **Target**: 13.1
	13. Municipal official plans	**Goal 11—Sustainable Cities and Communities** **Targets**: 11.a, 11.b, 11.5, 11.6, 11.7 **Goal 13—Climate Action** **Target**: 13.1
**Expenditures**	14. Eco-Roof Incentive program 15. Clean Water and Wastewater Fund 16. Green Infrastructure Phase II 17. Investing in Canada Infrastructure Program 18. Natural Climate Solutions Fund 19. National Disaster Mitigation Program	**Goal 11—Sustainable Cities and Communities** **Targets**: 11.a, 11.b, 11.5, 11.6, 11.7 **Goal 13—Climate Action** **Target**: 13.1
**Regulation**	20. Great Lakes Protection Act 21. Nutrient Management Act	**Goal 15—Life on Land** **Targets**: 15.1, 15.2, 15.3, 15.4, 15.5, 15.9
	22. Planning Act	**Goal 11—Sustainable Cities and Communities** **Targets**: 11.a, 11.b, 11.5, 11.6, 11.7 **Goal 15—Life on Land** **Targets**: 15.1, 15.2, 15.3, 15.4, 15.5, 15.9
	23. City of Toronto Act 24. Municipal Act 25. Ontario Build Code	**Goal 11—Sustainable Cities and Communities** **Targets**: 11.a, 11.b, 11.5, 11.6, 11.7
	26. Toronto Green Standard	**Goal 11—Sustainable Cities and Communities** **Targets**: 11.a, 11.b, 11.5, 11.6, 11.7 **Goal 15—Life on Land** **Targets**: 15.1, 15.2, 15.3, 15.4, 15.5, 15.9
**Taxation**	27. Development charges 28. Municipal property taxes 29. Municipal stormwater fees	**Goal 11—Sustainable Cities and Communities** **Targets**: 11.a, 11.b, 11.5, 11.6, 11.7
**Public Ownership**	30. Infrastructure for Jobs and Prosperity Act	**Goal 15—Life on Land** **Targets**: 15.1, 15.2, 15.3, 15.4, 15.5, 15.9
